# Dynamic failure characteristics and acoustic emission response mechanism of coal mass with various coal burst liabilities

**DOI:** 10.1371/journal.pone.0313910

**Published:** 2025-04-09

**Authors:** Zhijie Zhu, Peng Wang, Fei Lv

**Affiliations:** 1 College of Mining, Liaoning Technical University, Fuxin, China; 2 Shaanxi Reignwood Yadian coal industry Co., LTD Yadian coal mine, Xi’an, China; Akita University: Akita Daigaku, JAPAN

## Abstract

Coal burst liability is a key influencing factor for the occurrence of impact rock burst disaster. Uniaxial compression tests were conducted on coal bodies with various coal burst liabilities to analyze the dynamic failure characteristics of typical coal bodies, dimension (fractal dimension is a measure to describe the irregular shape of complex body), and acoustic emission (AE) energy characteristics after the coal and rock failure under loading and to investigate the failure characteristics of coal bodies with various coal burst liabilities and their AE response laws. The results show that (1) the stronger the coal burst liability, the more transient and intense the failure, the faster the kinetic energy of the crushed particles, and the more apparent the brittle failure characteristics, (2) the fractal dimension values of the crushed particles of the coal specimens have a positive correlation with coal burst liability; the stronger the coal burst liability of the coal body, the higher the degree of specimen fragmentation, and the larger the fractal dimension value, and (3)the cumulative AE energy of coal bodies with various coal burst liabilities increases significantly and evolves in a “step” pattern before the stress approaches its peak. As the coal burst liability of the coal body changes from zero to strong, the cumulative AE energy experiences a longer calm period, the width of the “step” becomes narrower, and the stored energy of the coal body gradually shifts from phase dissipation to instantaneous release. The correlation between AE parameters and coal burst liability was investigated to provide an experimental basis for AE monitoring and early warning of coal or rock fracture and rock burst.

## 1 Introduction

The coal-rock medium is a complex heterogeneous material. When it is loaded, the energy accumulated inside it suddenly releases and propagates outward in the form of elastic waves, resulting in acoustic emission (AE) phenomenon [1]. Acoustic emission monitoring technology is an important means of underground rock burst monitoring and prevention [2–4]. In order to explore the damage law of coal and rock mass, the acoustic emission monitoring technology is used to identify and warn the internal damage of coal and rock, which provides a reasonable basis for the prediction and prediction of coal and rock burst.

Several studies have focused on the AE characteristics of the coal failure process and the relationship between coal damage and AE. Wu et al. [5] studied the dynamic damage characteristics of coal by using acoustic emission monitoring technology. Zhang et al. [6] revealed the correlation between the characteristics of AE parameter changes and rock bursts based on rock uniaxial compression AE tests, combined with linear elastic energy criterion analysis. Sun et al. [7] conducted uniaxial compression and AE tests on two common rock samples with strong rock burst propensity and discussed the relationship between mechanical characteristics, AE characteristics, and rock burst liabilities during rock failure and the evolution of fractal characteristics of AE parameters. Kong et al. [8] selected rock samples from the original cracks for uniaxial compression loading tests and found that the AE count values and cumulative AE count trends could qualitatively explain the damage evolution pattern of the loaded rock samples. Zhang et al. [9] quantified rock damage by acoustic emission events and energy, and concluded that acoustic emission energy can more accurately characterize and predict the damage of limestone with rock burst liabilities. Liu et al. [10] conducted experimental research on the damage evolution and acoustic emission characteristics of uniaxial compression coal rock, and concluded that the acoustic emission characteristics of coal rock can better describe its deformation and damage evolution characteristics. Wang et al. [11] research on the damage evolution of internal cracks based on acoustic emission, and the study of acoustic emission response law and crack evolution characteristics can well reflect the deformation, stress and crack development law of coal and rock mass. Zhang et al. [12] found that the change of parameters is the macroscopic characterization of the transformation process of coal-rock specimens from local failure to overall failure based on the acoustic emission event location experiment of coal-rock uniaxial compression. Zhang et al. [13] believed that the characteristics of rock materials have an important influence on the cumulative acoustic emission ringing count and energy. Li et al. [14] carried out acoustic emission tests on coal bodies with various coal burst liabilities, and concluded that there were significant differences in ringing count, energy and b value during the failure process of coal bodies with various coal burst liabilities. Yang et al. [15] carried out uniaxial acoustic emission test of coal with strong, weak and non-impact coal burst liabilities. The results show that the number of acoustic emission events increases gradually with the increase of coal burst liabilities. Jang et al. [16] found that the higher the water content of saturated rock specimens, the lower the frequency characteristic value of acoustic emission key points, and the greater the damage degree of specimens. Zhao et al. [17] found that under the same fracture scale, the energy release of shear cracks exceeds that of tensile cracks, and the duration and rise time of acoustic emission signals of rock failure are larger. Hou et al. [18] found that the cumulative count of acoustic emission is proportional to the initial confining pressure before the accelerated failure stage of sandstone events. Zhang et al. [19] divided the acoustic emission events under cyclic loading conditions into short key, medium key and long key, among which the medium key acoustic emission events have the highest correlation with the damage degree of coal samples. Zhao et al. [20,21] conducted an experimental study on the distribution of spatial AE events during rock fracture in different rock samples using AE monitoring and location techniques. Yuan et al. [22] analyzed the fractal characteristics of the distribution of spatial AE events during the failure process of rock specimens. Li et al. [23] used fractal analysis to study the AE characteristics of coal samples with defects at different inclination angles. Wu et al. [24] analyzed the relationship between rock characteristics and laboratory experiment characteristics under numerical simulation and field working conditions. Shkuratnik et al. [25] studied the AE characteristics of coal rocks under uniaxial compression. Wang et al. [26] investigated the AE spectrum characteristics during the deformation and fracture of coal rocks.

The above studies on AE mainly focus on the evolutionary characteristics of AE parameters and identifying precursor information of conventional coal or rock fractures while paying less attention to the correlation between AE and coal burst liability. therefore, to address this issue, we conducted uniaxial compression tests on coal bodies with various coal burst liabilities, analyzed the dynamic characteristics of the failure processes of coal bodies with various coal burst liabilities, fractal dimension characteristics after loaded crushing, and AE energy characteristics. Investigated the correspondence between the AE characteristics and the failure process to explore the correlation between the AE signals and coal burst liability, and provided an experimental reference for AE monitoring and early warning of coal or rock fracture and rock burst.

## 2 Uniaxial compression loading test of coal

### 2.1 Specimen fabrication

**Fig 1:**
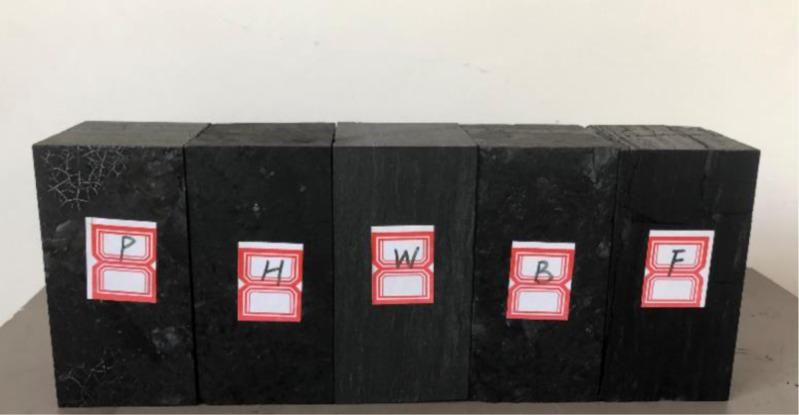
Some of the coal specimens.

In this paper, the coal specimens of typical coal mines such as Jinchuan Coal Mine in Xinjiang, Yadian Coal Mine in Shaanxi and Yanbei Coal Mine in Gansu are selected for testing (Table 1).with lumps of coal immediately wrapped with a cling film after retrieval from the site to ensure that the coal specimens were fresh and free from weathering or erosion. In order to ensure the uniform stress of rock samples in all directions, the machining accuracy is strictly in accordance with the standards of the International Society of Rock Mechanics.The specimens were processed into sizes of 50 ×  50 ×  100 mm, with the top and bottom surfaces polished to achieve an unevenness within 0.05 mm. A total of 12 samples were prepared and divided into 5 groups, Fig 1 shows the actual images of some of the coal specimens.

**Table 1:** The source and industrial analysis of different kinds of coal.

Mine name	Sampling location	Moisture content/%	Coal ash/%	Volatile fraction/%	Density/(g.cm^-3^)
Jinchuan Coal Mine	8 Coal seam E8104 working face	0.63-2.75	4.11-9.29	38.71-49.75	1.32
Yadian coal mine	4 Coal seam 1402 working face	2.41-6.26	8.65-33.87	28.58-38.94	1.38
Yanbei coal mine	5 Coal seam 250203 working face	5.33-13	3.85-17.71	35.59-41.93	1.42

### 2.2 Test equipment and test program

**Fig 2:**
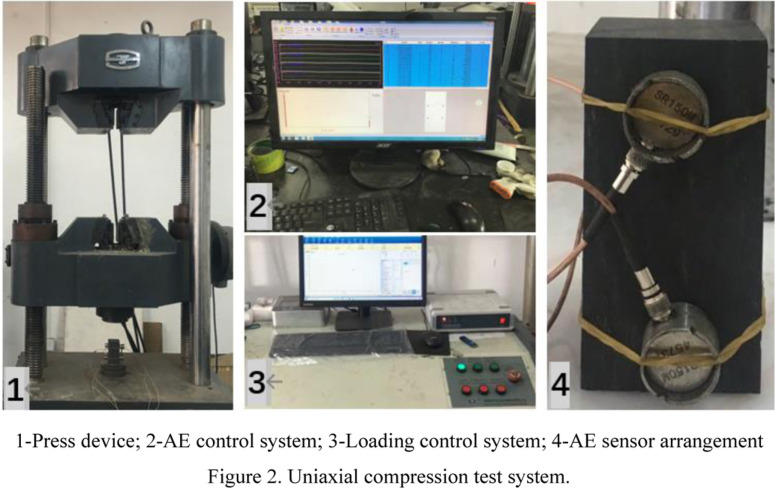
Uniaxial compression test system.

Uniaxial compression tests were performed on the WAW-600C electro-hydraulic servo rock mechanics test system, as shown in Fig 2, comprising a testing machine, a control system, and a stress-strain collector, characterized by high stiffness, good stability, and high measurement accuracy. The control system allowed for stress and displacement control modes during loading. The SAEU3H AE monitoring system (Qingcheng Acoustic Emission Research Company, Guangzhou), comprising four sensors, four preamplifiers, an AE host, an AE collector, and data processing software, was adopted as the AE monitoring system. The acoustic emission sensor is fixed on both sides of the coal body. Two sensors are arranged on the left side 30 cm away from the upper and lower boundaries, and the right side and the left side are symmetrically distributed to achieve real-time monitoring of AE characteristic parameters and the spatial location. An appropriate amount of Vaseline was applied between the sensors and the coal specimens (fixed with rubber bands), with the coupling degree of the sensors examined in lead break tests to ensure their coupling effect. Three analog signal sources are emitted at a distance of 100 mm from the sensor, and the response degree is measured respectively. The parameters of the acoustic emission signal are observed and analyzed to ensure the coupling effect. The AE sampling threshold was set to 45 dB, preamplifier gain: 40 dB, sampling frequency: 1 MHz, and number of channels: 4 to minimize external noise interference.

The test was conducted in the axial displacement loading control method, with a loading rate of 0.3 mm/min. During the compression of the specimens, a video recorder placed at 1 m from the facade of the specimen was utilized for video monitoring to record the deformation and failure images of the specimens. The data acquisition system collected force and displacement data in real time and monitored the loading pro-cess and AE synchronously. After the coal specimen failed, loading and monitoring were halted, experimental data were recorded, and the test bench was cleaned.

## 3 Experimental result analysis

### 3.1 Determination of coal burst liability

For discriminating coal burst liability, rock mechanics laboratory tests were conducted to obtain basic data, and then the relevant evaluation indexes are calculated.. The coal burst liability criteria in Table 2 are based on two basic parameters—bursting energy index and uniaxial com-pressive strength. Based on relevant data acquired from the test, the results of various discriminatory indicators were calculated and listed in Table 3.

**Table 2:** Criteria for bursting energy index and uniaxial compressive strength coal burst liability.

Discriminatory method	Calculation formula	Coal burst liability level classification		Notes
Bursting energy index	K_E_ = As/Ax	None	<1.5	As: Pre-peak cumulative deformation energy; Ax: Post-peak cumulative deformation energy
		weak	1.5–5	
		strong	>5	
Uniaxial compressive strength/MPa	Rc	None	<7	/
		weak	7–14	
		strong	>14	

**Table 3:** Discrimination results of specimen coal burst liability.

Coal specimen	Uniaxial compressive strength		Bursting energy index		Actual propensity level
	Size/MPa	Coal body propensity	Size	Coal body propensity	
H-2	4.7	None	1.3	None	None
H-1	6.6	None	1.1	None	None
P-2	12.5	weak	1.6	weak	weak
B-2	16.1	strong	4.8	weak	weak
B-1	13.73	weak	2.3	weak	weak
W-2	11.49	weak	2	weak	weak

**Fig 3:**
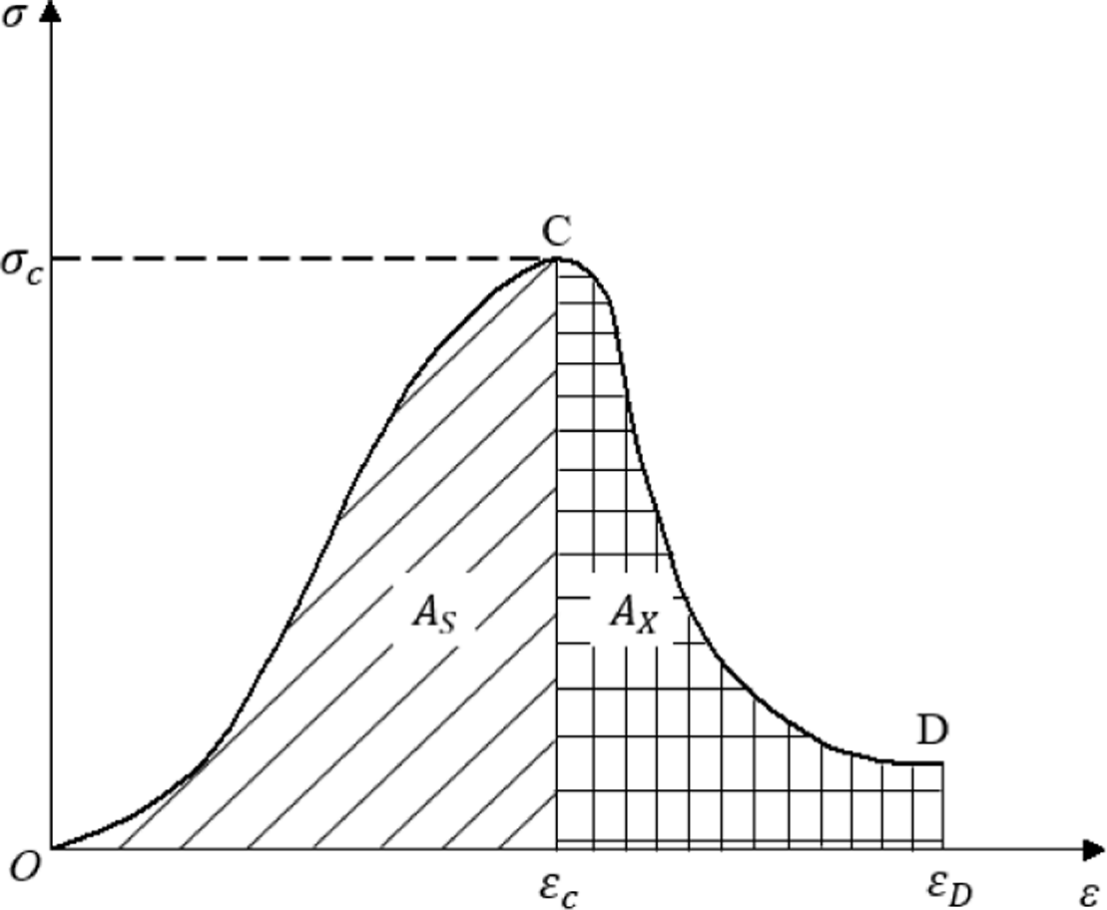
Schematic diagram of bursting energy index calculation.


The bursting energy index KE refers to the ratio of the cumulative pre-peak strain energy AS to the post-peak strain energy AX for the whole process stress-strain curve of the coal specimen. In Fig 3, C and D are the peak uniaxial compression strength and residual strength of the coal specimens, respectively. The pre-peak and post-peak strain energies were separately calculated in the area integration method based on the following equations:

$$A_{S}=\overset{\varepsilon_{C}}{\underset{O}{\int }}\sigma d\varepsilon$$ (1)

$$A_{X}=\overset{\varepsilon_{D}}{\underset{C}{\int }}\sigma d\varepsilon$$ (2)

where $\varepsilon_{C}$ and $\varepsilon_{D}$ are the strain values corresponding to the peak strength and residual strength, respectively. Based on the above equations, the bursting energy index of the specimens is expressed as:


$$K_{E}=\frac{A_{S}}{A_{X}}$$ (3)

### 3.2 Physical characteristics of coal

**Fig 4:**
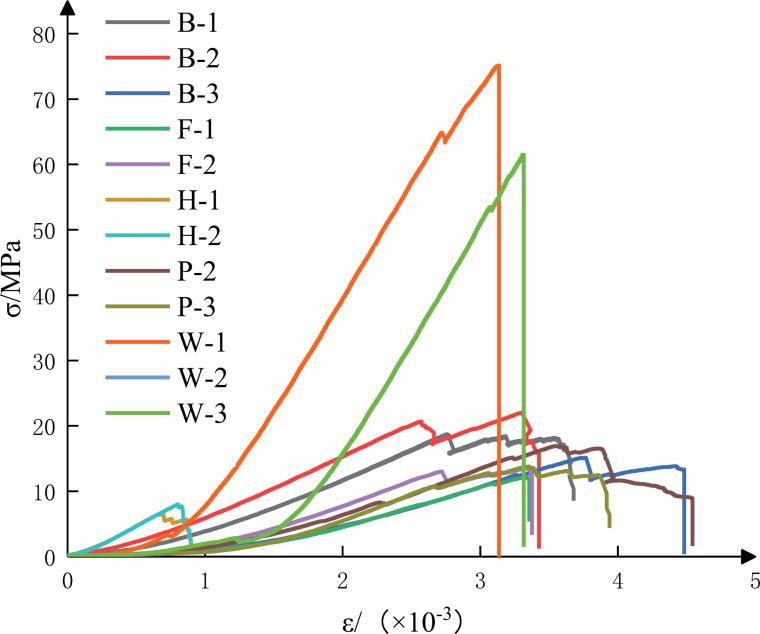
Stress-strain curve of coal body specimen.

The stress-strain curves of the 12 coal specimens are shown in Fig 4, which shows that the uniaxial compressive strength of specimen W-1 has the highest value and that of specimen H-2 has the lowest value. 12 coal specimens have compression-density stage, elastic deformation and stable damage stage, unstable rupture stage and post-rupture stage. Compared with the other coal specimens, specimen W-1, W-3 and F-2 have a long elastic deformation and stable rupture phase, and the unstable rupture phase is less obvious. After the peak point, the stress value drops rapidly, showing a sudden brittle damage, and the specimen undergoes a violent fragment ejection phenomenon, which has a typical strong coal burst liability. In contrast, the H-1 and H-2 specimens show an obvious unstable rupture stage before the damage, in which the fracture expands further and plastic deformation occurs, and the stress decreases slowly after the peak, and can be loaded until the bearing capacity is 0, with a complete full stress-strain curve, and no debris ejection after the damage of the specimen, which does not have the coal burst liability. The rest of the specimens showed weak local debris ejection during the damage process and had weak coal burst liability characteristics.

### 3.3 Analysis of the dynamic failure characteristics of typical coal bodies with various coal burst liabilities

#### (1) Coal specimen without coal burst liability.

**Fig 5:**
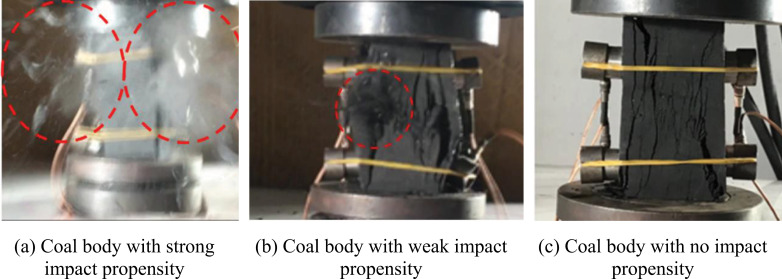
Dynamic failure diagram of coal specimens with various coal burst liability.

The dynamic failure of the coal specimen without coal burst liability is shown in Fig 5(a). After the stress reached the peak value, cracks appeared on the surface of the coal body, which extended and penetrated to the upper and lower surfaces; the coal flakes tended to fall off on the left side of the specimen, making a slight sound. The coal specimen produced only fractures during the whole damage process, without debris ejection, and the failure process, mainly tensile failure, lasted for a long period.

#### (2) Coal specimen with weak coal burst liability.

The dynamic failure of the coal specimen with weak coal burst liability is shown in Fig 5(b). When the stress reached the peak value, the cracks on the surface of the coal body continued to derive and develop, extending to the upper and lower surfaces. The specimen completely failed, with a small amount of debris ejected(As shown in the red circle in the picture), accompanied by severe noise. The coal specimen failure process lasted for a long period, but the degree of failure was minimal and plastic failure characteristics were apparent.

#### (3) Coal specimen with strong coal burst liability.

The dynamic failure of the coal specimen with strong coal burst liability is shown in Fig 5(c). The coal body failed significantly when the stress reached its peak value. The failure process was brief, accompanied by a bursting sound. Coal debris was ejected in all directions at high speed, covering a far distance and accompanied by thick smoke(As shown in the red circle in the picture). Except for a small amount of energy dissipated by plastic deformation, such as pore fracture compression, the energy of the coal specimen in this type before the peak was stored in the coal body as elastic strain energy; thus, the most energy accumulated before the peak was elastic strain energy. The brittle characteristics of the post-peak failure were apparent, with considerably small strain energy permanently dissipated in plastic deformation. The cumulative elastic strain energy of the coal specimen was instantaneously released as kinetic energy under considerably small deformation, which explains the phenomenon of coal debris ejection in all directions at high speed.

### 3.4 Characteristics of fractal dimension after failure of coal bodies with various coal burst liability

Crushed particles of coal bodies have rich information, and their fractal dimensions have wide applications. The study of the fractal dimension can be used to reflect the degree of the coal body crushing under load and investigate the influence of the coal burst liability factor on the degree of the coal body crushing. In this study, the fractal dimension D was calculated using the crushed particle mass—equivalent particle size, calculated as follows [27–29]:


D=3−a (4)

$$a=\frac{\text{lg}\left(\frac{M_{L}}{M}\right)}{\text{lg}L}$$ (5)

where a is the slope value in the log-log plot of l $\text{lg}\left(\frac{M_{L}}{M}\right)$ - lgL, *L* is the characteristic size of the equivalent particle size in the statistical interval, *M* is the total mass of crushed particles, and *M*_L_is the mass of crushed particles with the equivalent particle size less than *L*.

The apertures of the classifying screens selected in this study were 0.075, 0.25, 0.5, 1, 2.5, 5, 10, 20, 30, and 50 mm, in ascending order of size. For particles larger than 20 mm, the length, width, and thickness of the coal briquettes should be measured several times with a vernier caliper and the average value must be taken. Finally, the sieved crushed particles were weighed and calculated based on equations (4) and (5). Researchers [30] found that the fractal dimension D of crushed particles in the local range is larger than those in the overall range, with a higher correlation coefficient *R*2. To calculate the fractal dimension uniformly, the upper and lower fractal threshold values were set to 50 and 0.075 mm, respectively.

**Fig 6:**
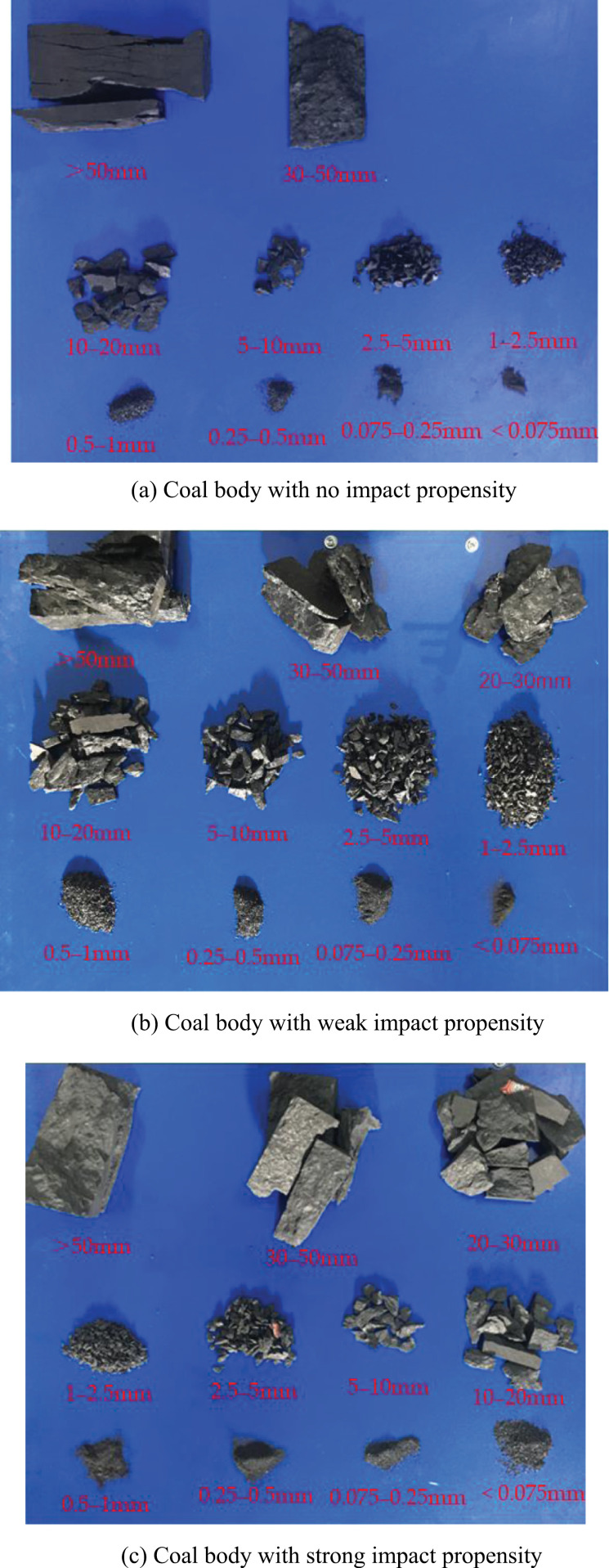
Distribution of sieved particles in typical coal bodies with various coal burst liabilities.

Fig 6 shows the distribution of multi-particle sizes of crushed particles of typical coal specimens with no, weak, and strong coal burst liabilities, respectively. Based on the figure, (1) crushed particles of coal specimens without coal burst liability with particle sizes > 50 mm have a larger volume and greater mass, with no particles within the particle size of 20–30 mm. The small number and mass of crushed particles with sizes < 20 mm indicate that coal specimens without coal burst liability exist in the form of large-sized particles after crushing. (2) The number of crushed particles with sizes > 30 mm of coal specimens with weak coal burst liability is small, with two pieces of crushed particles present in each size range of 30–50 mm and > 50 mm. Compared with coal specimens without coal burst liability, crushed particles of coal specimens with weak coal burst liability exist in all particle size ranges after crushing and have a more uniform mass distribution, indicating that the crushed particles after the coal body failure show a gradual transition from large size to small size with the increase in the coal burst liability of the coal body. (3) Only one particle with size > 50 mm and low mass is present after crushing the coal specimen with strong coal burst liability, but a larger number of small-sized particles are present, whose mass accounts for a high proportion, which indicates a significant reduction in the size of the large fragments and a concomitant reduction in mass, with the fragments gradually converging to a similar mass.

**Fig 7:**
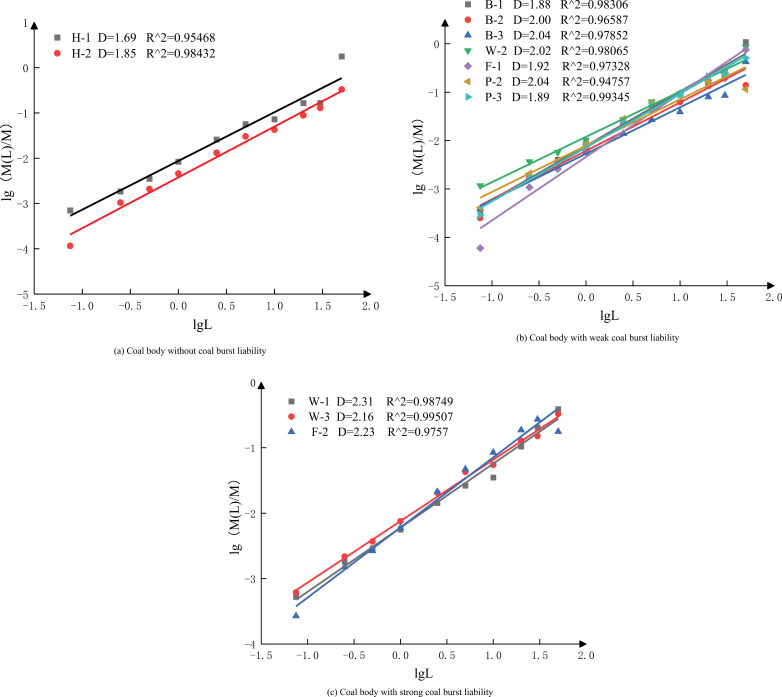
lg (M(L)/M)-lgL curve of coal bodies with various coal burst liabilities.

Table 4 lists the mass distribution of crushed particles of each coal specimen in each characteristic size range after being sieved. Fig 7 shows the fractal curves and fractal dimension values of coal bodies with various coal burst liabilities. Based on Fig 7, the correlation coefficients R2 of all 12 coal specimens are greater than 0.95, indicating a significant fractal characteristic of the coal specimens and a good linear correlation of the double log $\text{lg}\left(\frac{M_{L}}{M}\right)$ - lgL. If the measured data are linearly correlated in several different good segments, their distribution would have statistical self-similarity at multiple scales. According to this method, the fractal dimension is D =  0–3. When D =  2, the proportion of the fragment mass in each size range is equal. When 0 < D < 2, the proportion of the fragment mass in the large size range is higher. In contrast, when 2 < D < 3, the proportion of fragment mass in the small size range is higher.

**Table 4:** Mass distribution of crushed particles of coal specimens.

Coal specimen	Corresponding mass/g of particles smaller than the characteristic size L									
	0.075 mm	0.25 mm	0.5 mm	1 mm	2.5 mm	5 mm	10 mm	20 mm	30 mm	50 mm
H-2	0.02	0.18	0.36	0.79	2.27	5.25	7.37	15.28	22.2	118.73
H-1	0.01	0.18	0.43	1.04	3.87	8.67	15.69	29.41	41.95	65.76
F-1	0.08	0.21	0.4	0.95	2.93	6.43	8.24	18.74	18.74	56.76
B-1	0.04	0.21	0.47	1.03	3.05	6.5	10.5	15.89	29.88	49.59
P-3	0.1	0.6	1.2	2.63	8.34	18.79	30.24	73.62	83.32	125.36
B-2	0.09	0.63	1.21	2.65	7.62	15.2	22.49	56.06	70.26	182.29
P-2	0.18	0.91	1.66	3.69	12.33	26.8	40.08	71.7	103.85	175.5
B-3	0.17	0.73	1.18	2.37	5.95	11.54	16.8	34.13	36.7	318.96
W-2	0.3	0.94	1.46	2.63	7	16.26	23.67	40.85	50.27	112.77
W-3	0.21	0.74	1.26	2.57	6.99	14.6	18.7	43.79	51.36	200.98
F-2	0.1	0.56	0.99	2.22	7.88	17.55	31.49	69.45	100.99	140.99
W-1	0.19	0.66	1.05	2.03	5.15	9.5	12.65	37.58	73.82	218.56

According to the fractal dimension values of coal specimens, the fractal dimension values of coal specimens without coal burst liabilitity (H-2, H-1) are lower than 1.85, the fractal dimension values of coal specimens with weak coal burst liability (B-1, P-3, F-1, B-2, P-2, B-3, W-2) do not exceed 2.16, and the fractal dimension values of coal specimens with strong coal burst liability (W-3, F-2, W-1) are greater than 2.16. The value of fractal dimension D increases as the coal burst liability and the crushing degree of the coal body increase, with a critical value of fractal dimension D for each coal burst liability level.

### 3.5 Characterization of AE from coal bodies with various coal burst liabilities

**Fig 8:**
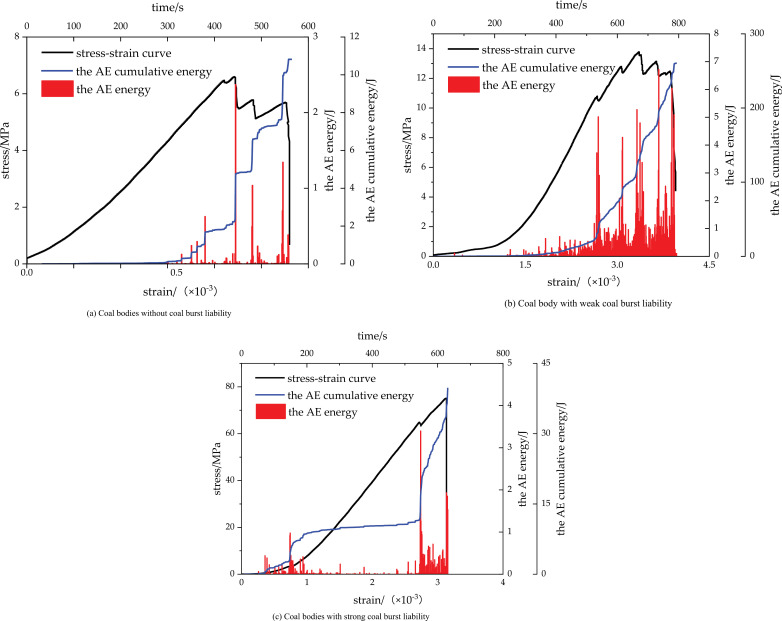
Curves of AE energy-cumulative energy–time-stress–strain relationship of typical coalies bodies with various coal burst liabilities.

AE activities, as a concomitant phenomenon of coal or rock deformation and failure, can effectively reflect the internal damage and evolutionary characteristics of coal or rock; their application to coal burst liability analysis can better reveal the mechanism of coal burst liability. In this study, the coal burst liability of coal specimens is comprehensively discriminated based on uniaxial compressive strength and impact energy index, classified into three levels: none, weak, and strong. Due to space limitations, typical coal specimens with various coal burst liabilities were selected, with curves of the AE energy-cumulative energy–time–stress–strain relationship of each specimen plotted, as shown in Fig 8. The variation characteristics of AE energy and the cumulative energy of coal bodies with various coal burst liabilities during loading are as follows:

(1) No coal burst liability: The cumulative energy of AE changes in stages during the loading process. Before the stress reaches 70% of the peak stress, the AE energy is low, and the cumulative energy growth is not significant. Thereafter, the cumulative energy shows an apparent increase as the energy of the single AE increases. The coal body is not immediately fractured when the stress reaches its peak value. The AE energy and cumulative energy continue to increase after the stress-strain curve fluctuates. The cumulative AE energy grows in a “step” pattern, with an arc section appearing between “steps,” indicating that micro-element failure occurs inside the coal body to produce microfractures during the loading process. In addition, the continuous increase in the cumulative AE energy indicates that the fractures in the coal body continuously expand and the energy stored within the coal body is gradually dissipated. Serious failure occurs inside the coal body and the cumulative AE energy sharply surges when the fractures penetrate each other to form a fracture plane.
(2) Weak coal burst liability: Before the stress reaches 70% of the peak stress, the AE occurs with low energy, and the cumulative energy appears to slowly grow over a long period, possibly related to the internal microstructure and microfracture of the coal body; moreover, the low energy indicates no apparent failure occurrence inside the coal body. Therefore, the cumulative AE energy during this period is still in a calm period. Between 70–100% of the peak stress, where the acoustic emission activity is violent, the cumulative energy sharply increases as the energy of single acoustic emission increases. The cumulative AE energy rapidly increases in a “step” pattern, with a certain angle between the “steps” instead of an arc section. Additionally, the width of the “step” becomes narrower, indicating that a large amount of energy is stored inside the coal body. In the late loading period, the internal structure of the coal body changes at different moments, resulting in the periodical release of energy. However, the time interval of energy release is shorter compared to that without coal burst liability, indirectly reflecting that the energy release from the coal body gradually transitions from periodical release to instant release as the coal burst liability of the coal body increases.
(3) Strong coal burst liability: Before the stress reaches 90% of the peak stress, the cumulative AE energy remains virtually unchanged, and the cumulative AE energy begins to sharply increase when approaching the peak stress. A small increase is still observed in the AE energy after the failure of the coal body due to the existence of residual strength of the coal body, and the damage occurs again under the stress. During the whole loading process, the change of cumulative AE energy only has one “step,” indicating that only one sharp increase occurs in the cumulative energy, appearing near the peak stress, indicating that the coal body with strong coal burst liability has stored great elastic strain energy in the early stage, whose instantaneous release causes the damage of the coal body. This result is precisely in line with the characteristics of coal bodies with strong coal burst liability (i.e., the instantaneous release of high-strain energy stored within the coal body).

**Fig 9:**
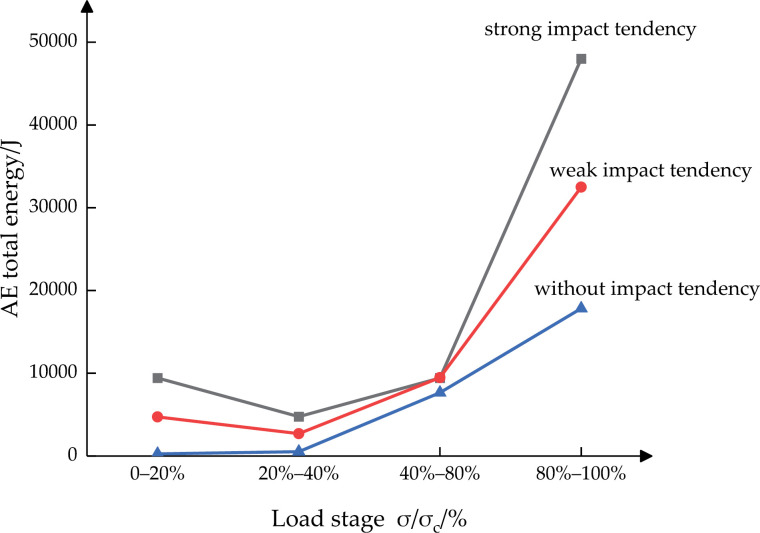
Characteristics of total AE energy at different stages of coal bodies with various coal burst liabilities.

According to the uniaxial compression fracture law of coal or rocks [31], the pre-peak failure process of coal bodies can be divided into four stages—initial compaction stage, elastic deformation stage, stable development stage of micro-fractures, and unstable development stage of fractures. The characteristic stresses corresponding to the different stages are fracture closure stress σ_cc_, fracture initiation stress σ_ci_, fracture penetration stress σ_cd_, and peak stress σ_c_, respectively. According to general experience [15], σ_ci_ is approximately 40% of the peak stress, while σ_cd_ is approximately 80% of the peak stress. Therefore, the pre-peak fracturing process of the coal body is divided into four stages—0–20%, 20–40%, 40–80%, and 80–100%—in terms of the ratio of the loaded stress to the peak stress. Moreover, the characteristics of the total AE energy of the coal body at each stage are quantitatively and statistically analyzed, as shown in Table 5 and Fig 9.

**Table 5:** Statistics of the total AE energy of the coal body at different stages.

Loaded stage σ/σ_c_/%	Total energy of coal body with strong coal burst liability/J	Total energy of coal body with weak coal burst liability/J	Total energy of coal body without coal burst liability/J
0–20	39434.90	4723.93	270.76
20–40	4765.00	2715.03	535.62
40–80	9432.31	9484.93	7661.79
80–100	47990.44	32496.20	17834.09
Cumulative energy/J	71622.66	49420.09	26302.27

According to Table 5and Fig 9, the total AE energy released during the loaded failure process of the coal body reflects the intensity of the coal burst liability of the coal body to a certain extent. Both the total AE energy of a single stage and the cumulative total AE energy increase with the enhancement of the coal burst liability, with the total AE energy of the coal body positively correlated with the coal burst liability.

From the characteristics of the AE energy evolution, the total AE energy of the coal body with strong and weak coal burst liabilities is the lowest at the second stage when the coal body enters the elastic deformation stage, where the coal body produces elastic deformation with a smaller number of microfractures. Subsequently, the total AE energy of the coal body shows a gradually growing trend, and the coal body enters the stable development stage of microfractures. Among them, the growth of AE energy of the coal body with strong coal burst liability is relatively lagging, and AE only starts to sharply increase after entering the fourth stage. The total AE energy of the coal body with weak coal burst liability starts to increase in the third stage, followed by a gradual and steady increase. The total AE energy of the coal body without coal burst liability shows a gradual and steady increase throughout the loading process, with the AE activity substantially active in the third stage.

By combining the characteristics of the total AE energy evolution, it can be concluded that the stronger the coal burst liability of the coal body, the more hysteretic the total AE energy activity and the shorter the waring time of coal body failure, suggesting a positive correlation between the amount of the total AE energy and the coal burst liability.

## 4 Conclusion

In this study, the dynamic failure characteristics, fractal dimension characteristics, and characteristics of cumulative AE energy of typical coal bodies were analyzed by conducting uniaxial compression tests by selecting coal specimens from coal mines and using both the rock mechanics test system and AE detection system. The main conclusions are as follows:

(1) The stronger the coal burst liability of the coal body, the more brief and violent the failure process, the faster the kinetic energy of crushed particles, and the more apparent the brittle failure characteristics.
(2) The fractal dimension value of crushed particles of coal specimens has a positive correlation with the coal burst liability of the coal body (i.e., the stronger the coal burst liability of the coal body, the higher the degree of fragmentation of specimens and the larger the fractal dimension value).
(3) Before the stress approaches its peak, the cumulative AE energy of coal bodies with various coal burst liabilities increases significantly and evolves in a “step” pattern. As the coal burst liability of the coal body changes from zero to strong, the cumulative AE energy experiences a long calm period, the width of the “step” becomes narrower, and the stored energy of the coal body gradually shifts from phase dissipation to instantaneous release.

(4) This paper mainly explores the influence of coal burst liabilitie on the dynamic fracture characteristics of coal and acoustic emission signals. The relevant research results provide experimental basis for acoustic emission monitoring and early warning of coal rock fracture and rock burst. The research method can provide reference for the subsequent research on the coal burst liabilitie of coal samples.

## Discussion

Although this paper has preliminarily explored the failure characteristics and acoustic emission laws of coal specimens with different coal burst liabilities, there are still the following problems, which need to be further studied.

(1) Coal and rock mass has its unique occurrence characteristics, physical and mechanical properties. There are great differences in mine samples with different stress levels and geological conditions. The damage characteristics of coal and rock mass with different coal burst liabilities under different mine geological conditions are explored, which can effectively guide the prediction of coal and rock dynamic disasters.
(2) Temperature and humidity have an important influence on the mechanical properties of coal and rock. The compressive strength of coal and rock decreases with the increase of temperature and increases with the increase of humidity. The failure characteristics of coal and rock at high temperature are obviously different from those at room temperature, and the increase of humidity alleviates the failure of coal and rock.
(3) The uniaxial compressive strength and elastic modulus of the coal-rock combination are larger than those of the coal sample. The current test does not consider the damage characteristics of the coal-rock combination with different impact tendencies, which is also a direction for future research.
(4) The failure characteristics and fractal dimension characteristics of coal and rock mass play a guiding role in the fracture of free face of coal and rock roadway. All kinds of precursor information before coal failure can be used as early warning of coal and rock fracture. Acoustic emission ringing count reflects the development of internal cracks in coal and rock mass, and early warning of failure from the inside of coal and rock mass. How to combine existing technologies with early warning systems needs further research.
